# Phenotypical-genetic virulence and resistance in atypical avian pathogenic *Escherichia coli*

**DOI:** 10.1007/s10123-026-00811-6

**Published:** 2026-04-02

**Authors:** A. L. da Silva, F. S. Luna, J. F. Nogueira, D. S. Rosa, P. I. de Figueirêdo, B. A. S. da Cruz, J. J. S. Gouveia, B. Brenig, M. M. da Costa

**Affiliations:** 1 Universidade Federal Rural de Pernambuco (UFRPE), Rede Nordeste de Biotecnologia - Renorbio, Recife, Brazil; 2 Animal Husbandry Department, Universidade Federal do Vale do São Francisco (UNIVASF), Petrolina, Brazil; 3 Institute of Veterinary Medicine, Georg-August-University Goettingen, Goettingen, Germany

**Keywords:** APEC, Colistin resistance, *E. coli*, MDR, Poultry

## Abstract

Avian pathogenic *Escherichia coli* is a threat to poultry industries, as it is associated with infections, poor production ratings, and economic losses. The spread of multidrug-resistant bacteria is a major factor aggravating the situation. Therefore, this study aimed to characterize 28 *E. coli* isolates from poultry farms in Brazil regarding virulence, as well as genetic and phenotypical resistance. Antimicrobial susceptibility test was carried out by the disk diffusion method, while the genomic information of the isolates was determined by whole-genome sequencing. Resistance was identified for all 19 antimicrobials tested, except imipenem. Elevated resistance was obtained for rifampicin (96.4%) and oxacillin (67.9%); intermediate resistance for the carbapenems (ertapenem, meropenem, 10.7%), and tetracyclines (doxycycline, 10.7%) was also detected. Genotypic analyses showed that the most common resistance genes were *tetA* (39.3%) and *gyrA* (39.3%). Remarkably, the plasmid-mediated gene, *MCR-1.1*, associated with colistin resistance, was detected in two isolates. The isolates tested did not carry the typical avian pathogenic *E. coli* genetic virulence panel; however, they presented great genetic diversity with prominent pathogenic potential connected to mechanisms of adhesion (*fimH*, 96.4%), immune evasion (*ompA* and *ibeB/C*, 100%; *traT*, 92.9%), and iron acquisition (*febB*, 100%; *iroN*, 60.7%). The overlap between genotypic and phenotypic information emphasizes the health risk imposed by such atypical strains and their dispersion capability amongst both animals and humans. Our findings contribute to a better understanding of the diversity of avian pathogenic *E. coli* and reinforce the importance of combined diagnostic and surveillance strategies in the control of such bacteria.

**Supplementary Information:**

The online version contains supplementary material available at 10.1007/s10123-026-00811-6.

## Introduction

One of the most important sanitary problems that aviculture currently faces is the occurrence of colibacillosis, a disease caused by avian pathogenic *Escherichia coli* (APEC) that can, in many cases, lead to significant economic and zootechnical losses (Denamur et al. 2021; Barros et al. 2025). These *E. coli* strains are a real threat to intensive production systems, as they are capable of causing local and systemic infections, especially in young chicks, which often leads to mortality and low production ratings (Ha et al. 2025; Jhandai et al. 2025).

Moreover, in recent decades, the issue of the spread of multidrug-resistant *E. coli* has become a matter of particular concern and is considered a major challenge due to its potential to undermine the effectiveness of conventional antimicrobial therapy, making the control of such infections difficult, and increasing the risk of spreading resistance genes not only to other animals but also among humans (Hasan et al. 2022; Ha et al. 2025).

Another concern is the ability of *E. coli* strains to develop antimicrobial resistance independently, as well as to acquire and exchange virulence and resistance genes through plasmids, transposons, and other mobile genetic elements with other bacteria, thereby promoting the sharing and persistence of such genes (Dawadi et al. 2021; Barros et al. 2025). Consequently, poultry products may act as reservoirs of MDR APEC and other pathogenic strains, enabling their transmission to humans (Dawadi et al. 2021; Ha et al. 2025).

The present work aims to characterize the genotypical and phenotypical profile of *E. coli* isolates from poultry farms in Santa Catarina, Brazil, analyzing both in silico and in vitro data, to try to gain a deeper understanding of the mechanisms connected to the occurrence of colibacillosis outbreaks.

## Materials and methods

### Bacterial isolates

28 lyophilized *E. coli* isolates were donated by a veterinary diagnostic laboratory, Mercolab, having been previously obtained from colibacillosis outbreaks on poultry farms in Santa Catarina, Brazil. The isolates were collected at different times between 2018 and 2022, distributed as follows: 2018 (4 isolates), 2019 (6 isolates), 2020 (6 isolates), 2021 (5 isolates), and 2022 (7 isolates). Upon arrival at our laboratory, the isolates were registered in the National System of Genetic Resource Management and Associated Traditional Knowledge (SisGen, no. A4E44C8). A standard strain, *E. coli* ATCC 25,922, was used as a control for the phenotypic analysis.

### Phenotypic characterization

#### Isolation and identification of *E*. *coli*

*E. coli* isolates were rehydrated and cultivated in MacConkey agar, and further identification was performed through additional biochemical tests (Markey et al. 2013). Confirmed *E. coli isolates* were stored in Brain Heart Infusion (BHI) broth with 40% glycerol, at -20 °C for subsequent molecular and antimicrobial resistance analyses.

#### Antimicrobial susceptibility test

Susceptibility to antimicrobials was determined by disk diffusion method, according to the recommendations of the Clinical and Laboratory Standards Institute (CLSI 2023). 19 antibiotics, representing the following classes of antimicrobials, were used: β-lactams (penicillins (ampicillin 10 µg, oxacillin 1 µg), cephalosporins (cephalexin 30 µg, cefotaxime 30 µg), and carbapenems (ertapenem 10 µg, imipenem 10 µg, meropenem 10 µg), aminoglycosides (gentamicin 10 µg, amikacin 30 µg), fluoroquinolones (ciprofloxacin 5 µg, enrofloxacin 5 µg), folate pathway inhibitors ((sulfamethoxazole–trimethoprim 30 µg), phenicols (chloramphenicol 30 µg, florfenicol 30 µg), nitrofurans (nitrofurantoin 100 µg), quinolones (nalidixic acid 30 µg), tetracyclines (tetracycline 30 µg, doxycycline 30 µg), and rifamycins (rifampicin 5 µg). The results were determined by the diameter (in mm) of the inhibition zones and isolates were classified as “susceptible” (S), “intermediate” (I), or “resistant” (R) to the evaluated antimicrobials (CLSI 2023).

The multiple antimicrobial resistance index (IRMA) was calculated based on the number of antimicrobial classes to which each isolate was resistant, divided by the total number of antimicrobials tested (*n* = 19), as adapted from the method described by Krumperman (1983). The class in which at least one antimicrobial exhibited a resistance profile was considered resistant. Results classified as intermediate were not considered for the calculation of the index.

### Genotypic characterization

#### Genomic DNA extraction

Total DNA was extracted using the commercial HiYield™ Genomic DNA Mini Kit (Real Genomics™), following the manufacturer’s instructions. DNA concentration and purity were determined by spectrophotometry (NanoDrop^®^), and integrity was verified by electrophoresis on 1% agarose gels.

#### APEC genome sequencing

Whole genome sequencing (WGS) of *E. coli* isolates was performed through the Illumina MiSeq next-generation sequencing (NGS) platform. Libraries for whole-genome sequencing were prepared using Nextera XT DNA Library Prep Kit (Illumina^®^), in accordance with the standard protocol. DNA fragmentation and adapter addition were performed simultaneously through enzymatic tagging. A dual index was incorporated into each sample for multiplexing during sequencing. Sequencing was performed on the Illumina MiSeq platform using the v3 reagent kit, generating 300 bp (2 × 300 bp) paired-end reads. A minimum coverage of 30× was defined to ensure the reliability of the assemblies for subsequent bioinformatic analysis. Quality control on the raw FASTQ files was completed using FastQC. And residual adapters were removed with the Trimmomatic tool before *de novo* genomic assembly and subsequent analytical steps.

#### In silico analysis

The genomic sequences of the 28 isolates were obtained by paired-end sequencing and subjected to *de novo* assembly using Shovill software. Each assembly was executed with four threads, and the generated contigs were organized individually by sample. QUAST (version 5.3.0) was used to evaluate the quality of the assemblies, and the results were summarized into a single report by the MultiQC software (version 1.32). For the automated functional annotations of the genomes, Prokka software version 1.15.6 was used. Information on the sequencing locus and center was added, and the functions for detecting conserved RNAs and families were enabled. The screening of antimicrobial resistance genes was performed using two distinct and well-established approaches: the complete VFDB database and VirulenceFinder, both executed via ARIBA v.2.14.7, applying strict identity and coverage criteria. Using RGI v.6.0.5 (Resistance Gene Identifier), the annotated protein files (.faa) were processed against the CARD database, in protein mode, with eight threads and automatic removal of intermediate files. In parallel, DeepARG v.1.0.2 software was used with the LS model to identify genes in nucleotide sequence (.ffn), applying a minimum probability of 0.8 and alignment identity of at least 30%.

Additionally, the ARIBA package was used for simultaneous screening of resistance genes (argannot bank), plasmids (PlasmidFinder), and virulence factors (VFDB and VirulenceFinder), with the banks previously prepared and updated. ARIBA analyses were performed from the raw fastq files, with ten threads per sample. MLST typing was performed using two independent methods: (i) through ARIBA, using *Escherichia coli* #1 and #2 schemes extracted from PubMLST, and compiling the results with the compileMLST.py script; and (ii) with the mlst command, using the contig files as input for cross-validation of the allelic profiles.

Lastly, the results obtained from the gene annotation were converted into a binary matrix, which was then analyzed using the R environment, and analysis was conducted with the pheatmap library (v.1.0). Further information, such as the Multilocus Sequence Typing (MLST) data and the origin of the isolates, was included for the graphical analysis, which allowed for the generation of heatmaps for the visualization of the genetic data obtained from the isolates.

## Results

### Phenotypical susceptibility to antibiotics

**Fig. 1:**
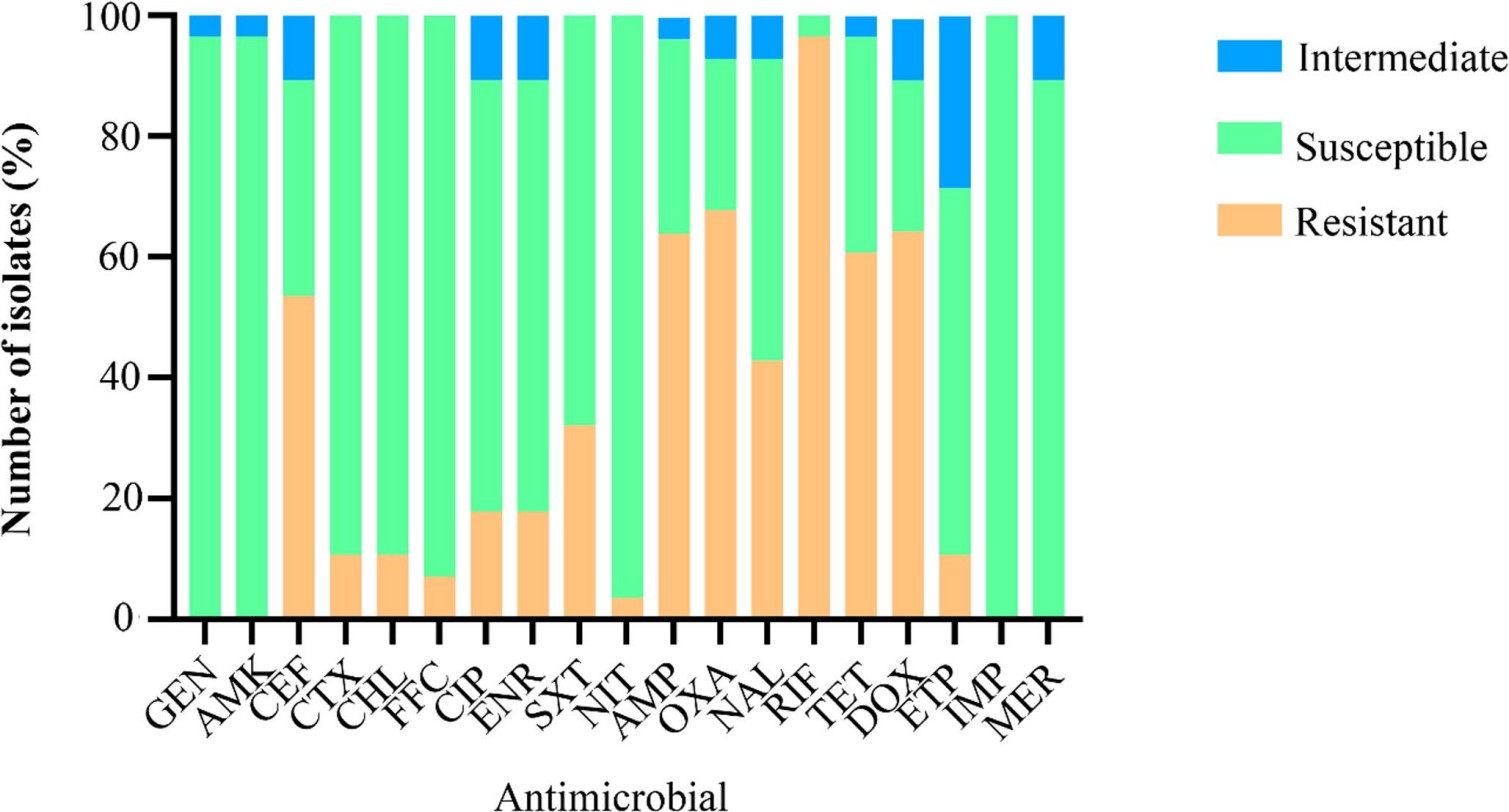
Phenotypic antimicrobial susceptibility profile of *Escherichia coli* isolates obtained from chickens on poultry farms. Bars represent the percentage of isolates classified as susceptible (green), intermediate (blue), or resistant (orange) to each antimicrobial agent tested, according to standardized interpretive criteria (PRISMA v8.0 2025)

Different levels of resistance were observed for all 19 antimicrobials tested, with the exception of imipenem. Based on the susceptibility profile (Fig. 1), the highest resistance rate was observed for rifampicin (96.42%) and oxacillin (67.85%). In contrast, the lowest resistance levels were recorded for florfenicol (7.14%) and nitrofurantoin (3.57%). The presence of intermediate resistance was more evident among carbapenems (ertapenem 10.71% and meropenem 10.71%) and tetracyclines (doxycycline 10.7% and tetracycline 3.57%).

**Fig. 2:**
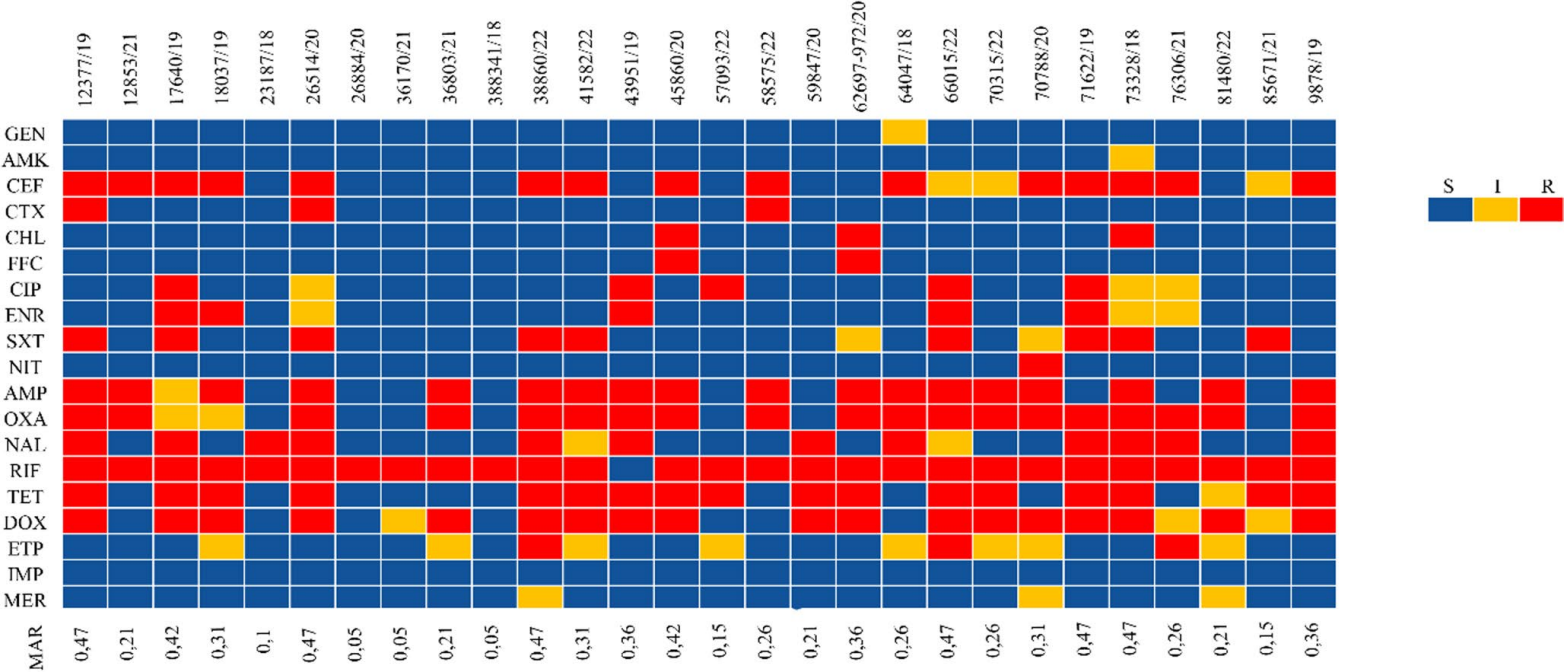
Heatmap of Antimicrobial Resistance Profiles and IRMA Values of the *Escherichia coli* Isolates. Rows represent antimicrobial agents and columns represent bacterial isolates (Microsoft Excel 2024). Red blocks indicate resistance (R), blue blocks indicate susceptibility (S), and yellow blocks indicate intermediate activity (I). Antimicrobials evaluated: GEN (gentamicin), AMK (amikacin), CEF (cephalexin), CFX (cefotaxime), CHL (chloramphenicol), FFC (florfenicol), CIP (ciprofloxacin), ENR (enrofloxacin), SXT (sulfamethoxazole–trimethoprim), NIT (nitrofurantoin), AMP (ampicillin), OXA (oxacillin), NAL (nalidixic acid), RIF (rifampicin), TET (tetracycline), DOX (doxycycline), ETP (ertapenem), IMP (imipenem) and MER (meropenem)

Moreover, it was possible to identify that 100% of the *E. coli* isolates in this study exhibited resistance to at least one of the antimicrobials tested. In addition, based solely on IRMA values, 75.86% (22/28) of the isolates presented indices above 0.2 and were therefore classified as multidrug-resistant (MDR), as shown in Fig. 2. These isolates demonstrated concomitant resistance to a high number of the antimicrobials tested. High IRMA values indicate extensive diversity of antimicrobial classes to which these isolates are resistant, suggesting strong selective pressure in the environment of origin and strengthening the potential clinical and epidemiological risks presented by these bacteria, as it shows significantly reduced therapeutic options.

### Genotypic virulence and resistance

Several antimicrobial virulence and resistance genes were observed in the isolates analyzed. Due to the large volume of data, the complete results are available in the supplementary material (Table S1). Genome assemblies had sizes ranging from 4.78 to 5.65 Mbp, consistent with the expected genome size of *E. coli*. N50 values varied between 85 Kbp and 707 Kbp, with most assemblies above 150 Kbp. The largest contigs reached up to 1.24 Mbp, and L50 values were low, indicating overall reliable assembly quality for genomic analyses.

#### Virulence factors

Out of the 28 isolates, nine isolates simultaneously presented the four classic virulence markers of the APEC panel – *iutA*,* hlyF*,* ompT*, and *iroN*, reinforcing their pathogenic potential as shown in Table 1. In addition, all of them carried genes with functions related to complement system evasion and survival in serum, such as *traT*,* ompA*, and *ibeB*.

**Table 1:** Most relevant virulence genes identified among the 28 *E. coli* isolated from poultry

Functional Category	Gene	Frequency among isolates (%)
APEC panel (core genes)	*hlyF*	71.42%
	*iroN*	60.71%
	*ompT*	57.14%
	*iutA*	42.85%
Adhesion	*fimH*	96.42%
Iron acquisition	*febB*	100%
	*sitA*	71.42%
	*iro*	60.71%
	*chuA*	42.85%
	*iucA*	42.85%
Immune evasion	*ompA*	100%
	*ibeB/ibeC*	*100%*
	*traT*	92.85%
	*ompT*	57.14%
Toxins	*hlyE*	85.71%

In spite of its importance in pathogenic *E. coli* strains, such as APEC, the presence of the *iss* gene was not detected in any of the isolates examined. The absence of this gene in all the analyzes carried out indicates that this gene may not be part of the genomes evaluated.

A few isolates (*n* = 3) carried only three out of the five genes traditionally used as molecular indicators of APEC, rendering them beyond the most restrictive diagnostic limits for this group. However, each carried a number of other relevant virulence genes, such as adhesins (*fimH*,* papC*), siderophores (*iucA-D*,* fepA-D*,* chuA*), and immune evasion factors (*traT*,* ompT*), contributing to the pathogenic potential of these isolates.

#### Resistance factors

**Fig. 3:**
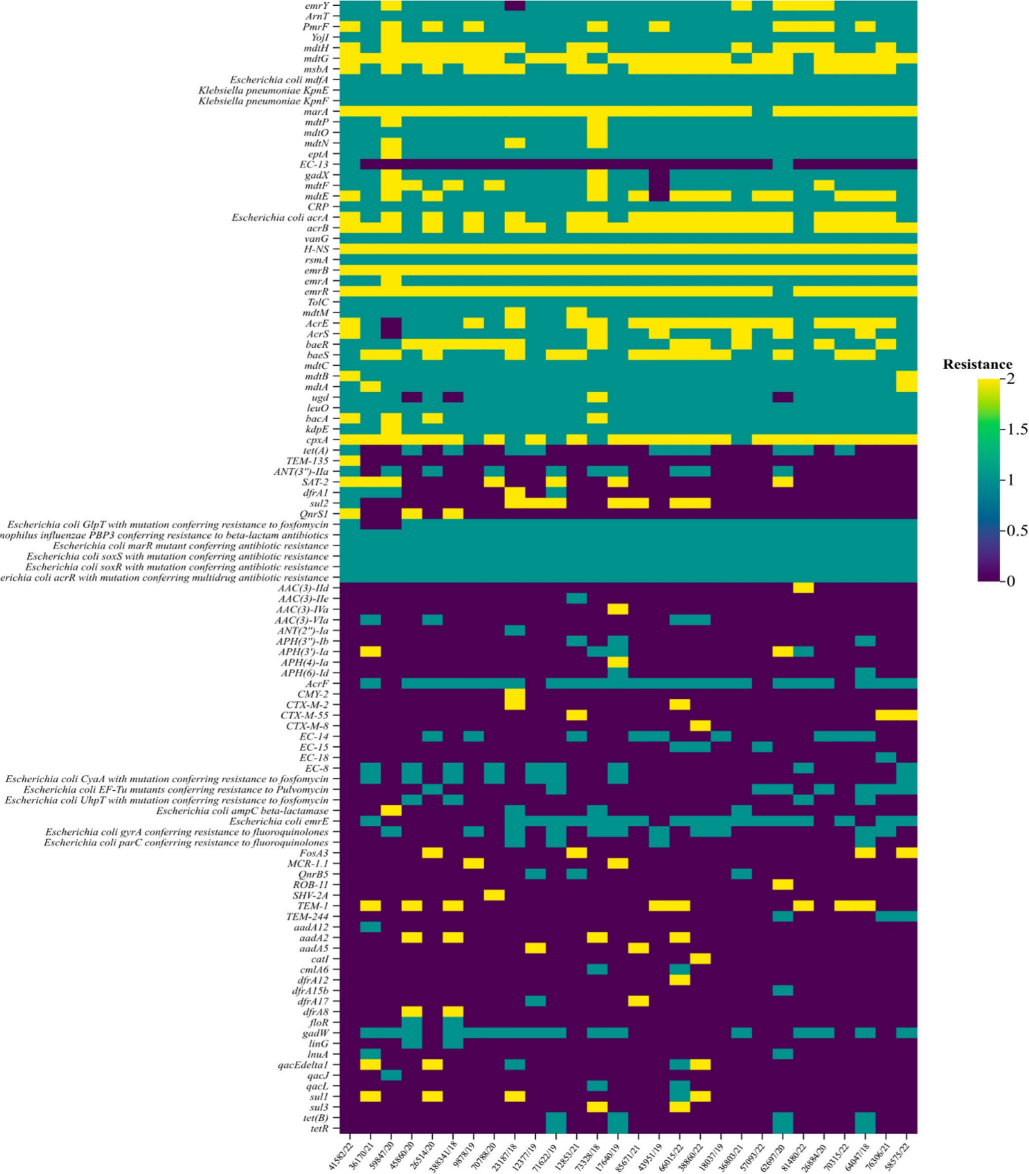
Distribution and expression of resistance genes across all 28 isolates, according to Resistance Gene Identifier (RGI) (ChiPlot v7.0 2025). Each row corresponds to a gene, while columns represent individual isolates

Similar to what was observed regarding virulence, the presence of numerous resistance genes was also remarkable. All genes detected are showcased in Fig. 3, as well as the intensity of their expression.

ESBL genes were present in some isolates, the most frequent being *CTX-M-55* (10.7%), *CTX-M-2* (7.1%), and *CTX-M-8* (3.6%), all of which were a part of the *CTX-M* operon. *TEM* gene variants were also observed in 12 isolates out of the 28 isolates (42.9%), and the most frequent one was *TEM-1* (28.6%), followed by *TEM-244* (10.7%) and less frequently *TEM-135* (3.6%). *AmpC* was present in 4 isolates (14.3%).

For aminoglycoside resistance genes, the majority were detected. *ANT(3’’)-IIa* gene was the most prevalent, detected in ten isolates (35.7%). Other genes of significant frequency included *APH(3’)-Ia* (17.9%) and *aadA2* (14.3%). Some forms of the acetyltransferase members of the *AAC(3)* family were also found, although not as commonly. The quinolone resistance genes were also found, namely *gyrA* (39.2%) and *parC* (14.3%), *qnrS1* (10.34%) and *qnrB5* (10.3%).

13 out of 28 isolates (46.4%) contained sulfonamide resistance genes, the most prevalent being *sul2* (28.6%) and *sul1* (14.3%), while *sul3* was present in a single isolate (3.6%). As far as tetracycline resistance genes are concerned, they occurred in 12 out of 28 isolates (42.9%), with the *tet(A)* gene prevailing in 11 isolates (39.3%), followed by four isolates (14.3%) in which the *tet(B)* variant occurred, and the *tetR* regulatory gene in four samples. The *dfrA* variant responsible for resistance against trimethoprim was encountered in 11 out of 28 isolates (39.3%). The *dfrA1* was the most prevalent gene (17.9%). Other variants that were found included *dfrA8*,* dfrA12*,* dfrA15b*, and *dfrA17*. The presence of these genes, especially together with the *sul* genes, enhances the probability of resistance to the TMP-SMX therapeutic regimen.

Genes that encode resistance to phenicol were found in five isolates, with *cmlA6* and *floR* each found in two (7.1%) and *catI* found in one (3.6%). For the *MCR-1.1* gene related to colistin resistance, this was found in two isolates (7.1%).

MLST exhibited a diverse population of isolates, with 20 distinct sequence types identified. The most frequent were ST117 (*n* = 3), ST955 (*n* = 2), ST1673 (*n* = 2), and ST155 (*n* = 2). Other lineages included ST101, ST57, ST10, ST602, ST641, and ST349, among others, indicating substantial genetic diversity among the strains.

Concerning mobile elements, plasmid replicon typing revealed a wide diversity of incompatibility groups among the isolates. IncF-type plasmids were the most frequent, detected in many strains, while Col-type replicons were also common. Notably, IncI2 and IncX4 replicons were identified in some isolates (*n* = 1 and *n* = 3, respectively), which are known vectors of the *MCR-1* gene, consistent with our detection of colistin resistance. These findings reinforce the presence of multiple plasmid structures potentially involved in the dissemination of resistance and virulence determinants.

## Discussion

In the present study, we chose to highlight the genes considered most relevant from an epidemiological, clinical, and public health perspective, based on their frequency, association with multidrug resistance, or known role in pathogenicity.

### Virulence profile

Our genotypic analysis of the isolates exposed a broad range of virulence genes, with profiles ranging from classical lineages compatible with APEC to atypical strains carrying alternative determinants with comparable pathogenic potential. As previously noted, nine of the 28 isolates analyzed presented the four classical markers of the APEC panel (*iutA*,* hlyF*,* ompT*, and *iroN*) defined by Johnson et al. (2008), and therefore can be considered APEC. Although the five genes that compose the panel act as genetic markers for avian pathogenic *E. coli*, it’s important to note that the profile of such strains is not solely defined by the presence of these genes. As stated by Kazimierczak et al. (2025), APEC strains are highly diverse and may present several virulence markers, frequently including iron acquisition, adhesins, invasins, protectins, and toxin genes.

In this study, the APEC strains lacked the *iss* gene, traditionally associated with increased serum resistance (Tivendale et al. 2004). Although this may indicate a specific limitation in complement resistance, it appears to be compensated for by the combined action of other evasion genes such as *traT*,* ompA*, and *ibeB*, all associated with complement evasion and serum survival (Ikeda et al. 2021). The recurring nature of *cvaC*, which is involved in the production of microcin (Bhambure et al. 2024), may also provide a competitive edge in challenging environments such as the intestinal tract of birds.

Adhesion genes, especially those related to type 1 fimbriae (*fimA-fimH*), were also present at a high frequency, indicating the functional or almost complete operon in most strains. The presence of fimbriae-related genes is a common feature in APEC strains, as described in recent studies on colibacillosis by Jalil et al. (2023), Saidenberg et al. (2024), Bhambure et al. (2024), among others. These genes are responsible for the adhesion of the pathogen to the intestinal tract of the host, and their presence may indicate the horizontal acquisition of intestinal adhesion determinants (Foroogh et al. 2021), as described by the genomic plasticity of the strains in this study.

Immune evasion and tissue invasion ability were further enhanced by the presence of high numbers of *traT*,* ompA*, and *ibeB/ibeC* genes. Yet, the number of these genes is vastly different among *E. coli* populations and geographical areas, as observed in studies performed in China (Lu et al. 2022) and South Korea (Ha et al. 2025). The concurrency of these genes indicates an elevated potential for extracellular survival, systemic infection (Ikeda et al. 2021), and colonization of protected sites (Hasan et al. 2022).

Iron acquisition, a critical process for bacterial survival and a cofactor for various cellular processes (Zhang et al. 2025), was indicated by the presence of a wide and strong array of siderophore-related genes, including *iucA-D*,* chuA*,* sitABCD*,* iroBDE*,* febB-G*, and *iroN*. This array may contribute to host colonization and rapid bacterial growth (Spiga et al. 2023). In recent studies on avian *E. coli*, *iroN* and *iutA* are the most common iron acquisition-related genes, as part of the APEC panel, as reported by Meng et al. (2024) and Jhandai et al. (2025). However, as previously discussed, we observed a large diversity of iron acquisition genes in the present study. These findings indicate a broad, redundant, and adaptive siderophore capacity, consistent with systemically competent pathogenic profiles.

To summarize, these virulence factors provide an explanation for the mechanism of colibacillosis outbreaks. Adherence factors are important in colonization (Jalil et al. 2023; Bhambure et al. 2024), immune evasion factors are important in systemic spread (Ikeda et al. 2021), and iron acquisition is important in bacterial growth (Spiga et al. 2023). This combination of traits clarifies how atypical APEC strains can establish infection and trigger outbreaks, even in the absence of the classical APEC virulence factor set.

Additionally, the *hlyE* toxin, detected in the majority of the isolates, represents an additional virulence factor with important cytotoxic function. This finding goes against the findings of most studies with APEC strains, which generally do not report the presence of this toxin gene and instead describe the occurrence of *vat* (Awawdeh et al. 2024; Ha et al. 2025). In recent literature, the only work to report the occurrence of *hlyE* in APEC was conducted by Saidenberg et al. (2024), and similar to this study, it evaluated APEC strains from poultry in Brazil. Such findings could suggest that *hlyE* is more prevalent in South America. However, further studies are necessary to confirm this hypothesis.

### Phenotypical and genotypical resistance

Our findings reveal a widespread distribution of multidrug-resistant profiles combined with the presence of a genetic arsenal that strengthens this potential in avian *E. coli*, constituting a concerning scenario. These findings indicate that while the isolates are genetically diverse, certain lineages were recurrent, suggesting clonal dissemination. In particular, ST117 and ST10 are recognized sequence types associated with both avian and human infections (Saidenberg et al. 2024), emphasizing their epidemiological importance. The coexistence of diversity and clonality highlights the dual dynamics of resistance and virulence spread in atypical APEC populations, with implications for both poultry production and public health.

The results observed amongst the phenotypic and genotypic findings indicates that a significant proportion of the strains not only actively express resistance but also carry determinants that may perpetuate this profile through horizontal gene transmission mechanisms.

The high rates of resistance to traditional antimicrobials, including rifampicin, penicillins, and tetracyclines, not only confirm the weakening of traditional therapeutic efficacy in poultry production, but also suggests it is related to the presence of *TEM*,* CTX-M*, and *tet* resistance genes. The repeated patterns observed may suggest the existence of a selection pressure due to the prolonged use of antimicrobials in poultry farming.

In this study, rifampicin exhibited the highest resistance rate, an expected finding, since this drug is reported to have low efficacy against *E. coli* when used as monotherapy (CLSI 2023; Wang et al. 2023). Phenotypically, high levels of total and intermediate resistance were found for the tested isolates, especially for β-lactam-based antimicrobials. The resistance could be connected with the presence of β-lactamase resistance genes, such as *TEM-1*,* TEM-135*,* CTX-M-2*,* CTX-M-8*, and *ampC*, which are usually present in strains resistant to β-lactam-based antibiotic agents (Jalil et al. 2023), as well as variants such as *TEM* and *CTX-M*, found in penicillin-resistant strains (Ha et al. 2025).

Tetracycline resistance related genes, *tetA*,* tetB*, and *tetC*, was observed in the tested strains that were also phenotypically resistant to the aforementioned antimicrobials. Therefore, our results further emphasize the strong connection between the presence of *tet*-type genes and the occurrence of phenotypic resistance. However, the correlation between the presence of *sul*-type genes and the prevalence of phenotypic resistance to sulfonamides is not as strong (Liu et al. 2024; Meng et al. 2024).

Resistance to first-generation cephalosporins (cephalexin) was high in our isolates (over 50%), whereas resistance to third-generation cephalosporins (cefotaxime) was relatively low (10.7%). In human patients, however, WHO GLASS (2026) surveillance data indicates that *E. coli* resistance to third-generation cephalosporins in Brazil ranged from 16% to 32% between 2018 and 2023, reflecting a concerning increase of resistance in clinical settings. The phenotypic resistance in humans is largely driven by ESBLs such as *CTX-M*,* TEM*, and *AmpC*, the same mechanisms we identified at the genetic level in our poultry isolates. The overlap between genotypic findings in animals and phenotypic resistance in humans underscores the shared pathways of antimicrobial resistance and highlights the importance of integrated surveillance across sectors.

When comparing our findings with national data from human patients in Brazil during the same period, similar resistance trends emerge. A large-scale surveillance study performed by Pillonetto et al. (2021) which reported ciprofloxacin and sulfamethoxazole-trimethoprim among the most tested antimicrobials, with resistance rates of 29.8% and 32.1%, respectively, similarly to what we observed in the present work. Such results overlap with our poultry isolates, which also exhibited high resistance to fluoroquinolones and sulfonamides, and carried ESBL genes such as *TEM* and *CTX-M*. The parallel between animal and human data reinforces the concern about shared resistance mechanisms and the potential for cross-transmission of multidrug-resistant *E. coli.*

This study did not identify carbapenemase-encoding genes, yet, some level of phenotypic resistance was observed which might be connected to other resistance mechanisms such as the efflux pump mechanism that enables the regurgitation of substances (Rosa et al. 2024). *The MCR-1.1* gene that is responsible for colistin resistance was observed in two strains, even though colistin was not part of the antimicrobials tested, the identification of this gene is a public health concern due to the potential for the spread of the resistance provided by it through plasmids from chicken pathogens to human pathogens (Liu et al. 2024).

The detection of IncI2 and IncX4 plasmids among our isolates provides a plausible genetic basis for the presence of *MCR-1.1*. Both replicons have been reported as vehicles for the dissemination of colistin resistance across avian and human *E. coli* populations (Rodríguez-Martínez et al. 2021; Touati et al. 2025). This genetic evidence supports the presence of colistin resistance determinants even in the absence of phenotypic testing, highlighting the silent but significant risk posed by plasmid-mediated *MCR-1.1* dissemination. This highlights the importance of monitoring plasmid-mediated colistin resistance even in the absence of phenotypic confirmation. Their occurrence in atypical APEC strains highlights the potential for horizontal transfer of *MCR-1* between different lineages, supporting the epidemiological concern of plasmid-mediated colistin resistance. Together with the diversity of IncF-type plasmids carrying ESBL genes, these findings emphasize the role of mobile genetic elements in the co-selection and spread of multidrug resistance in poultry-associated *E. coli*.

Overall, our findings demonstrate that atypical avian *E. coli* strains combine diverse virulence factors with multidrug resistance genes. The parallel between genotypic profiles in poultry and phenotypic resistance observed in studies with human isolates highlights shared mechanisms of antimicrobial resistance.

## Conclusion

The virulence and resistance profiles of the tested isolates were highly diverse and revealed a concerning infectious potential. The phenotypical information analyzed showed that most isolates presented multidrug resistance, which was confirmed by the presence of genes such as *CTX-M*,* TEM*,* tet*, and *MCR-1.1*. Although the isolates did not present the standard APEC virulence markers, especially *iss*, they presented various other virulence genes associated with different methods of host colonization, adhesion, iron acquisition, toxicity and immune system evasion. Thus, these findings reinforce the atypical nature of these APEC strains and their potential to colonize, persist, and spread in different hosts. Our results highlight the importance of combined diagnostic and surveillance strategies and emphasize the health risk posed by these atypical strains.

## Supplementary Information

Below is the link to the electronic supplementary material.

Supplementary Material 1

Supplementary Material 2

## Data Availability

All data supporting the findings of this study are available within the paper and its Supplementary Information.
